# COVID‐19: Short‐Term Influence on China's Economy Considering Different Scenarios

**DOI:** 10.1002/gch2.202000090

**Published:** 2020-12-28

**Authors:** Wei Pan, Ge Huang, Yongdong Shi, Cheng Hu, Wan‐qiang Dai, Wulin Pan, Huang Rongsheng

**Affiliations:** ^1^ School of Applied Economics Renmin University of China Beijing 100872 China; ^2^ School of Economic and Management Wuhan University Wuhan 430072 China; ^3^ School of Business Macau University of Science and Technology Taipa 999078 China

**Keywords:** COVID‐19, economic impact, epidemics, scenario analysis

## Abstract

Recently, most countries have entered the outbreak period of the novel coronavirus epidemic. This sudden outbreak has caused a huge impact on the global economy, which has intensified the division of globalization and the recession of the global economy. Although the epidemic situation in China has gradually stabilized, the severe situation in the world still inevitably impacts China's economy. Based on the uncertainty of future epidemic, this paper sets up three scenarios to analyze the impact of the epidemic on China's economy. The first is that in June, the epidemic both at home and abroad is under control without rebound; the second is that the domestic epidemic is basically controlled but the foreign situation is not effectively controlled; the third is that the epidemic situation in China has a serious rebound due to the influence of the imported cases from abroad, which destroy the economy again. At the same time, some corresponding guidelines are put forward for the recovery of economy, and to minimize the economic losses as well as accelerate the pace of national economic recovery. In addition, it is believed that these suggestions may have certain reference value to other countries.

## Graphical abstract

Based on the uncertainty of the future of the current epidemic, this paper sets up three scenarios to analyze the impact of COVID‐19 on China's economy. At the same time, in order to minimize the economic losses as well as accelerate the pace of national economic recovery, some corresponding policy recommendations are put forward.

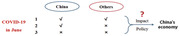

## 1 Introduction

Coronavirus disease 2019 (COVID‐19) is an infectious disease caused by severe acute respiratory syndrome (SARS) coronavirus,^[^ ^1^ ^]^ causing large‐scale infection worldwide. During this period, catering, tourism, entertainment, transportation and other industries have suffered huge loss, and the national economic development has been greatly affected in the short term. The effects of financial market weakness and the disruption to daily life around the world will trigger lower consumer spending and investment on top of the disruptions to the global supply chain.^[^ ^2^ ^]^ Research shows that the potential revenue loss of the air transport industry in the first quarter is about 4.1 billion dollars.^[^ ^3^ ^]^ Due to Chinese tours being canceled and a general recession in domestic and international travelers, Vietnam's tourist industry is expected to lose up to $7.7 billion in the first 3 months of this year.^[^ ^4^ ^]^ Thanks to the timely and effective prevention and control measures carried by our government and the desperate treatment of medical staff, the domestic epidemic situation has been basically stabilized, the number of newly confirmed cases and deaths reduced greatly, and people's lives are gradually on the right track. The next urgent problem to be solved is how to promote the enterprises to return to work as soon as possible, so that the pace of economic development in China can also be accelerated.

Compared with SARS, the key difference in this epidemic is that China's influence on the world has undergone a fundamental change.^[^ ^5^ ^]^ So lots of scholars have carried out relevant research to explore the impact of the epidemic on China's economy. Martin McKee and David Stuckler comment that if the world fails to protect the economy, COVID‐19 will damage health not just now but also in the future.^[^ ^6^ ^]^ Based on the input–output analysis, Duan H et al. estimated that sensitive industries such as tourism and entertainment may lose up to 18% of normal output.^[^ ^7^ ^]^ An and Jia made a simple comparison between this epidemic and SARS, sorted out current national response measures, and put forward some specific countermeasures and suggestions.^[^ ^8^ ^]^ Most scholars focus on fiscal and monetary policies. Li and Fu expressed the need of accelerate the construction to mitigate the impact of the epidemic.^[^ ^9^ ^]^ Based on the internal and external environment of the national economy, Cheng and Qian stressed that monetary policy should be implemented in advance.^[^ ^10^ ^]^ Zhang and Liu pointed out that the policy focus in 2020 is still on the dynamic balance between stable growth and stable leverage.^[^ ^11^ ^]^ Some scholars focus on specific industry areas, and have studied the impact of the epidemic on the development of the industry. Han and Zhu analyzed the operation difficulties of mining enterprises during the epidemic prevention and control period, and put forward several issues worthy of attention in future policies.^[^ ^12^ ^]^ Shen proposed rescue measures such as stabilizing foreign trade and investment to promote the recovery of foreign trade enterprises.^[^ ^13^ ^]^ Zheng et al. analyzed the impact of the epidemic on various consumption sectors and predicted the future consumption situation.^[^ ^14^ ^]^ Jiang put forward relevant policy suggestions on financial support by studying the impact mechanism of the epidemic on corporate financing.^[^ ^15^ ^]^ Kirigia et al. took research to estate the fiscal value of human lives lost due to COVID‐ 19 in China as of 24th February 2020, results show that the deaths from COVID‐ 19 had a discounted (at 3%) total fiscal value of almost $ 924 346 795 in China.^[^ ^16^ ^]^ Coherent and coordinated responses are needed the global policymakers to limit the economic fallout.^[^ ^17^ ^]^

In addition, although the situation in China has gradually improved, the international epidemic spread trend is still extremely severe, and the measures of blockade and epidemic prevention in various countries impact a lot on the world economy. According to the research report of the Oxford Economics, purchasing manager index (PMI) in the United States and Europe has reached a record low, and consumer confidence in South Korea has fallen back to the low point in 2009. It is estimated that the global gross domestic product (GDP) growth will shrink by more than 1% in 2020.^[^ ^18^ ^]^ According to the survey report of Institute for Supply Management, nearly 75% of companies have experienced supply chain disruption due to transportation restrictions.^[^ ^19^ ^]^ In the context of globalization, China, as an important part of the global industrial supply chain, will suffer huge losses during the outbreak of the epidemic. On the basis of considering the development of foreign epidemic situation, it is still of great practical significance for China's economic recovery to estimate the economic loss under different scenarios and to take effective measures to maintaining GDP growth of 6%.

## 2 Forecast of China's Economic Growth Without the Outbreak

In 2019, the overall situation of China's economy showed a rapid upward trend, the per capita GDP reached 70 892 yuan, which exceeded 10 000 US dollars for the first time (based on the annual average exchange rate), achieving a GDP of 99 086.5 billion yuan, a growth rate of 6.1%, the added value of the tertiary industry accounted for 53.9%. In addition, the total retail sales of social consumer goods exceeded 4 billion yuan, which showed the vigor and vitality of China's economy.

This paper uses the neural network model to predict the GDP of China in 2020 without epidemic. Neural network model is a kind of calculation model, which contains tens of thousands of connecting nodes, namely neurons. The calculation model of neural network is composed of these neurons, which are a kind of excitation function and realize the control of the output network through the bridge of different weights. The intelligent characteristic of neural network is particularly outstanding, showing strong self‐learning ability, self‐adaptive ability, and self‐organizing ability. Therefore, neural network has a very attractive prospect for the feasibility of future prediction. Based on the time series neural network algorithm, this paper forecasts the GDP over the years.

First, the normalization method of min–max is used to normalize the GDP data to the interval [0, 1], and the formula is as follows:

(1) $$\begin{array}{c} x^{*}=\frac{x\;-\;\min}{\max\;-\;\min} \end{array}$$

where max is the maximum value of sample data and min is the minimum value of sample data.

We get the time series of 2000–2019 as follows: *X* = [0    0.011 883 202   0.024 071 035   0.041 705 064   0.069 123 225   0.097 732 176   0.133 797 912   0.190 674 915   0.245 865 947   0.278 735 469   0.350 151 008   0.435 287 079   0.492 148 362   0.553 212 95   0.610 029 431   0.660 889 377   0.725 495 121   0.821 657 542   0.919 621 476   1].

**Figure 1:**
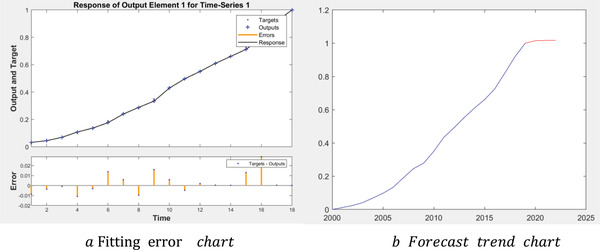
The fitting error chart and trend chart of China's GDP.

In this paper, the BP neural network is based on the *newff* function in MATLAB. Taking China's GDP data from 2000 to 2019 as the original data sample, the data of 2000 and 2001 are used to predict 2002, and then the data of 2001 and 2002 are used to predict 2003, and so on. The model is trained repeatedly until the absolute error between the fitted value and the real value is within the range of (−0.02, 0.02). Finally, we can get the error curve (**Figure** **1**a) and prediction trend curve (Figure 1b) between the simulated value and the real value. It can be seen that the maximum absolute error between the fitting value and the real value is −0.028 96, the minimum absolute error is 0.000 19, and most errors are between [−0.01, 0.01]. In addition, according to the predicted results, it is predicted that China's GDP in 2020 would be 104 902 billion yuan. In other words, it can be considered that China's GDP can reach 104 902 billion yuan in 2020 without the epidemic, breaking through the one billion mark for the first time, with a growth rate of 5.87%.

## 3 The Impact of the Epidemic on China's Economy

The PMI is usually used to measure the economic growth of various industries, and the line of expansion and contraction is 50%. Index higher than 50% represents the expansion of industry economy, lower than 50% means the decline of economy, index close to 40% indicates that it has the trend of economic depression, slightly greater than 50% means the economy is moving forward slowly, slightly less than 50% means the economy is moving toward recession slowly. The National Bureau of Statistics announced that the comprehensive PMI output index in February 2020 was 28.9%, 24.1% points lower than that in January.^[^ ^20^ ^]^ Among them, the manufacturing PMI was 35.7%, 14.3% points lower than last month, and non‐manufacturing business activity index was 29.6%, 24.5% points lower than last month.

In terms of sectors, affected by the epidemic, the upstream and downstream of the manufacturing supply chain are faced with different degrees of shutdown and production halt. In addition, the transportation is blocked, which leads to the slowdown even stop of the overall production and supply activities. In February, the manufacturing production index was 27.8%, down 23.% points compared with January. What is more, the residential segregation led to a significant contraction of market demand. The manufacturing new orders index and new export orders index in February were 29.3% and 28.7% respectively, down 22.1% and 20% points from January respectively. On the whole, due to the short‐term impact of the epidemic, the manufacturing sector faced a contraction of both supply and demand in February, making the manufacturing PMI drop to 14.3%.

The non‐manufacturing sector seems to suffers more. Similarly, from the perspective of supply and demand, the business activity index of non‐manufacturing industry in February 2020 is 29.6%, down 24.5% points from February; the new order index in February is 26.5%, down 24.1% points; the new export order index is 26.8%, down 21.6% points. The significant decline in supply and demand led to the overall market downturn, and even led to a significant decline in employment and raw material related indexes. In the non‐manufacturing industry, the construction industry is mainly affected by the shortage of workers and the blocked transportation of raw materials, which results in the delay of operation; the service sector is seriously frustrated in the epidemic due to its natural personnel aggregation and contact attribute, with the business activity index in February being 30.1%, 23.0% points lower than last month.

According to the relevant service industry data released by the National Bureau of Statistics, the information and Internet related service industry and financial industry suffer less, and the decline of the relevant industry index is relatively small. The business activity index of financial industry is 50.1%, which is still strong in the expansion range. The business activity index of telecommunication and Internet software industry benefits from Internet “cloud office” and other conditions, which are higher than the business activity index of service industry by 13.2% and 11.3% points respectively. However, the wholesale and retail industry affected by the supply of manufacturing industry declined significantly, the accommodation and catering, tourism, and transportation industry are experienced the hardest hit. Taking the catering industry as an example, the catering revenue from January to February was 419.4 billion yuan, down 43.1% year on year.

Overall, the epidemic will hit China's economy in the short term. In the first 2 months of this year, the total retail sales of social consumer goods reached only 5213 billion yuan, down 20.5% year on year in nominal terms (a 23.7% decrease in real terms after deducting price factors), and the investment in fixed assets (excluding rural households) fell 24.5% year on year. However, according to the information released by the National Bureau of Statistics on March 23, despite the impact of the epidemic on all walks of life, China's economy is still on a long‐term growth path. On the one hand, due to the Spring Festival, weather and other reasons, the main indicators of production, supply and demand, import and export, investment, and so on from January to February account for a small proportion of the total amount of the whole year. On the other hand, because the total economic volume of the second half of the year usually accounts for 55%, the first half of the year accounts for 45%, and the first quarter only accounts for about 20%. So as long as the economic recovery accelerates after the second quarter, we will have the opportunity to make up for the economic losses during January and February.

Academician Zhong Nanshan predicted that the epidemic could be basically controlled in June. Therefore, we take June as a turning point to analyze the impact of the epidemic on China's economy under three different scenarios: first, the epidemic is basically controlled at home and abroad in June, the supply chain is smooth, and the economy is fully recovered; the second is that the situation in China is basically controlled but the situation abroad remains uncontrolled; the third is that affected by imported cases from abroad, the epidemic situation in China appears a serious rebound phenomenon, shocking the whole economy again.

### 3.1 Scenario 1: The Epidemic Worldwide is Basically Controlled in June

Under the optimistic scenario, the epidemic situation worldwide can be basically controlled in June, and the impact on national economic activities and international trade would basically last until the end of the second quarter. Since March, the epidemic has been gradually controlled in most parts of China, the number of new cases in many areas has been remaining zero for several consecutive days. On March 13, the joint defense and joint control mechanism of the State Council released data that the average operating rate of industrial enterprises above a certain scale except Hubei Province exceeded 96%, and the economy is gradually recovering.

Since the blockade of Wuhan on January 23, 2020, all regions in Hubei Province have successively closed their channels until the end of March. Wuhan remove its blockade in April 8, 2020. Considering the economy contribution of Wuhan to Hubei Province, we conservatively set the suspension time of economic activities of Hubei Province as 2 months, that is, in the first quarter, the GDP of Hubei Province only reached 1/3 of the GDP without epidemic. In 2019, the GDP of Hubei Province reached 4582.831 billion yuan, increasing 7.5%, of which 911.005 billion yuan was completed in the first quarter, with an increase of 8.1%, accounting for 19.88% of the annual output value. Assuming the same growth in 2020, Hubei's GDP in the first quarter should be 984.786 billion yuan, and the annual output value should be about 4923.9 billion yuan. Affected by the epidemic, the output value of Hubei Province in the first quarter was about 328.2 billion yuan, down about 64% year on year.

Except for Hubei Province, the suspension time and degree of economic activities in all parts of the country in the first quarter are different. The economic slowdown in other parts of China during the epidemic period is not significant, this paper conservatively believes that the period of suspension of economic activities in the whole country (except for Hubei Province) caused by the epidemic is 15 days. In the first quarter of 2019, China's GDP reached 21 806.28 billion yuan, with a growth rate of 7.9%. With the same growth rate, the GDP of the first quarter of 2020 should be 23 528.98 billion yuan, and that of all regions except Hubei Province should be 22 544.184 billion yuan. Affected by the 15‐day suspension of economic activity, we estimate the output value of the country (except Hubei province) in the first quarter to be 18 786.82 billion yuan. The GDP in the first quarter of 2020 is 19 115.02 billion yuan, down about 12.34% year on year. Therefore, the GDP loss in the first quarter during the epidemic period would be about 4413.95 billion yuan.

According to the National Bureau of statistics, the output value in the first quarter usually accounts for about 20% of the annual output, 45% in the first half and 55% in the second half.^[^ ^20^ ^]^ As a result, the second quarter accounts for 25% of the national economy. We assume that China's economy will gradually recover to normal level in the second quarter according to linear changes, and then the output value in the second quarter will be 24 084.37 billion yuan, down 0.7% year on year. Considering that China's economic growth has always had the tradition of “ensuring 6,” the GDP in 2020 must reach more than 105 031.7 billion yuan, and the GDP in the third and fourth quarters must reach 61 832.3 billion yuan. In the third and fourth quarters of 2019, the output value is 53 022.84 billion yuan, the growth rate must reach 16.61% by 2020.

Generally, the first quarter is the peak consumption season. According to the estimation of the National Bureau of statistics, affected by the epidemic, the retail sales of social consumer goods decreased by more than 1.5 trillion yuan, the consumption of tourism, accommodation and other entertainment decreased by more than 1 trillion yuan, and the consumption demand suppressed by the epidemic was about 1.5 trillion yuan from January to February.^[^ ^20^ ^]^ These demands will be released gradually after the epidemic, and even the consumption rebound phenomenon would appear. In addition, relatively speaking, the impact of domestic import and export in the first quarter was not significant. In the first 2 months of this year, the total import value and export value decreased by 4% and 17.2% respectively year on year. Import was unaffected while export was slightly affected, but compared with the slowdown and stagnation of the overall domestic economy, it can be ignored. In combination with various situations, the state needs to issue relevant policies to stabilize economic growth from the perspective of consumption and investment, support small and medium‐sized enterprises, encourage enterprise production and stimulate overall consumption.

In order to ensure that the commercial enterprises can make up for the economic losses caused by the epidemic as soon as possible and quickly recover to the daily production and operation level, we propose three levels of short‐term support policies. The first is the inclusive preferential policies for all kinds of enterprises under general impact. Major financial and banking institutions can appropriately increase the credit line of small and medium‐sized enterprises, reduce the corresponding loan interest rate, or provide financing guarantee, so as to ensure that enterprises whose capital chain is broken due to the epidemic can get timely financial support. The second level is the incentive policies for enterprises that actively support the people's livelihood during the epidemic. The government may award certain material rewards and honors to enterprises that actively provide material support, avoid supply interruption and guarantee people's life during the epidemic period. The third level is the subsidy policies for the severely damaged tourism, catering, retail, and other enterprises. On the basis of the inclusive policy, we should increase the subsidy for such major damaged enterprises and set up special subsidy funds. The payment of real estate tax, urban land usage tax and other taxes can be appropriately postponed or reduced. At the same time, the bank enterprise consultation meeting may be held to realize the full connection between enterprises and banks or financial institutions. Through completely communication, the matching funding institutions can fully understand enterprises’ losses and specific needs, so as to provide effective targeted assistance to realize accurate and efficient matching between enterprise demand and subsidy supply.

In addition, investment and consumption have always been the main driving force for China's economic development. In terms of investment, first of all, we should focus on the smooth implementation of major projects to ensure the completion of the target tasks. Second, the government can attract social capital to participate in 5G base station, industrial Internet, artificial intelligence and other new infrastructure projects by expanding the scale of special debt, developing political credit finance, and other ways to ensure that infrastructure investment continues to play important role in stabilizing GDP growth. Stimulating residents to expand consumption is also a key way to enhance economy recovery. On the one hand, people's constrained and repressed consumption desire has gradually rebounded with the improvement of the epidemic situation; on the other hand, some regional governments have taken measures such as issuing consumption coupons to further stimulate the consumer end, which is undoubtedly a “strong shot” in the arm for enterprises.

However, the design of consumption coupons should be more scientific and reasonable, for example, the distribution object should focus on the poor and the distribution field should focus on catering, tourism, entertainment, and other impacted major enterprises of the people's livelihood, and the whole flow process of consumption coupons should be monitored and traced to ensure that consumption coupons can be spent in the short term, and the enterprises’ revenue in distress can be timely and fully stimulated. In terms of stimulating consumption, the government and enterprises can form a cooperative mechanism, in which enterprises give certain preferential treatment to consumers, and the government gives corresponding support to enterprises, so as to stimulate consumption in various aspects to drive economic operation.

In addition to the government's relevant subsidy policies, the self‐help measures of enterprises cannot be ignored. The impact of the epidemic has once again stressed the great potential and bright prospects of the Internet economy. Live e‐commerce has gradually become an important channel of transaction and communication channel between merchants and consumers. The amount of live transactions of BESTORE bucked the trend and increased by two times during the epidemic period. Therefore, enterprises should give full play to their subjective initiative, actively explore the digital transformation path, combine online channels with offline entities, and further expand their sales revenue by taking advantage of the characteristics of online economy such as openness, sharing, and lower cost. In addition, upstream and downstream enterprises in the industrial chain should help each other. Leading enterprises can provide financial support to enterprises in trouble by providing advance payment, payment of payables and other forms to relieve their cash flow pressure, so as to get through the industrial chain as soon as possible and restore the supply system in the whole chain.

### 3.2 Scenario 2: The Outbreak in China is under Control, But the International Situation is Severe in June

With the epidemic basically under control in late February and the resumption of work and production in many regions, most media believe that the outbreak will not have a huge impact on China's economy and will not change the long‐term stable and good situation of the economy. But this is based on the premise that the economy recovers steadily in the second quarter and the overseas epidemic will not affect the domestic economy. At present, the global situation has exceeded expectations. As of April 12, there are more than 1.5 million confirmed cases overseas, including more than 460 000 confirmed cases in the United States, and more than 100 000 cases in Spain, Italy, France, and Germany. In order to prevent the epidemic spreading, more than 20 countries, such as India, Poland and Canada, have announced to close their borders. Italy, Belgium, and other countries have implemented national closure, which will seriously affect the import and export of trade goods around the world, thus hindering the smooth flow of global supply chain, thus impact the economies of all countries.

In the second scenario, although the epidemic situation in China has been under control in the second quarter, the spreading of foreign epidemic situation has seriously affected China's import and export, and most of the domestic commodities have to be exported for domestic sale. In 2019, China's total import and export of goods reached 31.6 trillion yuan, an increase of 3.4% year on year. According to the forecast by the Prediction Science Research Center of the Chinese Academy of Sciences on January 8, 2020, it is estimated that China's GDP growth rate will be about 6.1% in 2020, among which consumption, investment, and net export will drive 4.4%, 1.4%, and 0.3% of GDP growth respectively, and the trade surplus is about $411.4 billion. According to the situation of previous years, there is little difference in the total value of import and export in each quarter. Suppose that the net export in the third quarter of 2020 accounts for 1/4 of the annual net export, that is, the GDP output value is about $102.85 billion.

According to statistics, affected by the domestic epidemic, the total value of China's exports from January to February is $292.45 billion, down 17.2% year on year; the total value of imports is $299.54 billion, down 4% year on year.^[^ ^20^ ^]^ If the global epidemic is not effectively controlled in June, it will be difficult for China's import and export activities to be carried out smoothly under the situation of multi‐national border blockade. Home isolation will reduce the total social demand. In the case of factory shutdown and residents staying indoors, the social demand will only be reflected in some basic protective materials and living materials such as masks, disinfectants, toilet papers, etc. The shutdown of factories will further lead to the fracture of the industrial chain, and the production of various commodities will be delayed, so China's import and export will continue to be affected in the third quarter. In the third quarter of 2019, China's total export value is $654.46 billion, import value is $535.38, resulting in a trade surplus of $119.08 billion. If the decline is 17.2%, the net export value in the third quarter of 2020 is estimated to be $98.6 billion due to the impact of the overseas epidemic. Compared with the above $102.85 billion, the net export loss in this scenario is about $4.25 billion, once again raising the difficulty of “ensuring 6” of the China's economy.

At present, there are many problems in the national foreign trade, such as the supply and marketing fracture, inventory accumulation, capital shortage, etc. The operation of foreign trade enterprises has great uncertainty. How to take precise and effective measures to reduce the impact of the global outbreak on the country's import and export and improve the efficiency of the resumption of cross‐border transactions is of great significance to stabilize the country's economic development.

We put forward the following foreign trade policy suggestions: first, in terms of financial and tax support, we should expand the scale of credit for foreign trade enterprises, launch special subsidy funds, appropriately cancel the guarantee and mortgage of foreign trade funds, strengthen tax relief, set up green channels for foreign trade to simplify business approval procedures, to solve the problem of capital shortage of foreign trade enterprises as soon as possible and effectively reduce the burden of enterprises. Second, in terms of market development, local governments, and foreign trade industry should actively cooperate to build a trade service platform and provide relevant trade demand, policy, and legal information around the world in a timely manner. Meanwhile, the government should encourage the full application of Internet of things, artificial intelligence, and other new technologies in business development, so as to enhance the competitiveness of China's foreign trade enterprises on the international stage.

### 3.3 Scenario 3: The Whole Country and Abroad are Affected by the Epidemic in June

**Figure 2:**
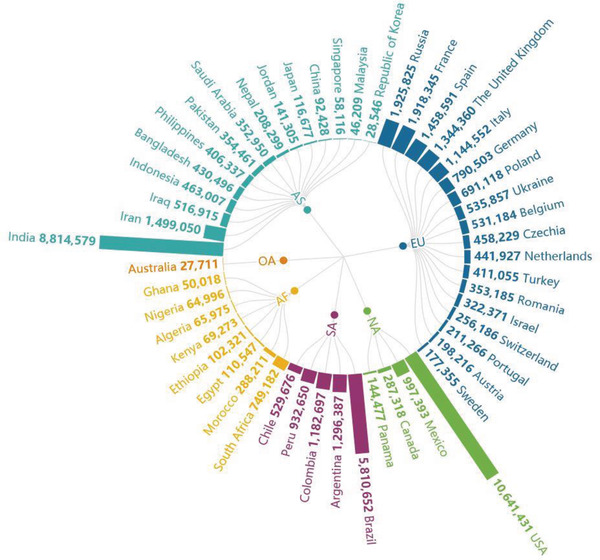
Total confirmed cases in some countries on 15 November, 2020.

**Figure 3:**
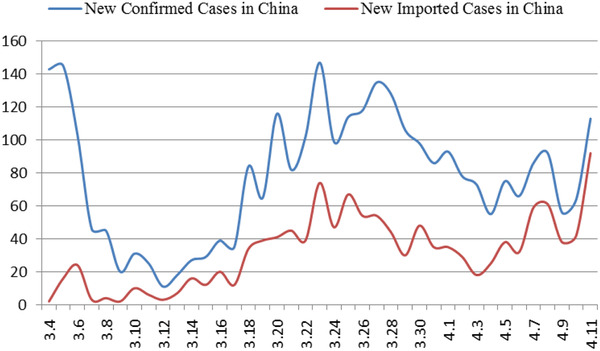
Trend of new confirmed/new imported cases in China.

**Figure 4:**
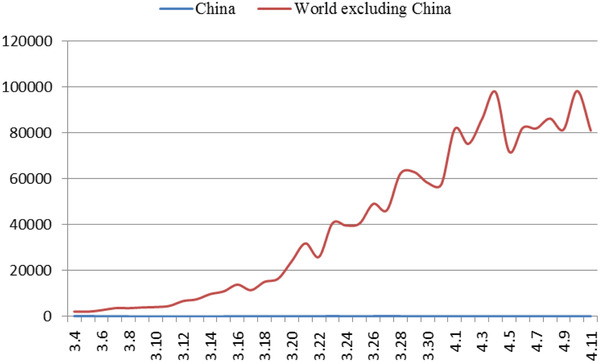
Trend of newly confirmed cases at home and abroad.

Since the middle of March, the epidemic situation in China has been basically controlled, but the international situation has spread (**Figure** **2**).^[^ ^21^ ^]^ While the domestic situation has stabilized, the number of confirmed cases abroad has increased sharply. As of April 7, the number of confirmed cases (number of confirmed cases = cumulative number of confirmed cases—cumulative number of cured cases—cumulative number of deaths) in China is 2229, and the number of confirmed cases in foreign countries exceeds 1 million (**Figure** **4**). The proportion of foreign imported cases in the daily new cases in China has been increasing gradually (**Figure** **3**). As of 24:00 on November 18, 2020, a total of 3735 cases had been imported from abroad.

In scenario 3, if the foreign epidemic situation does not get better in June, and China failed to take appropriate precautions such as proper control of entry and exit, the increasing number of imported cases is likely to lead to a large‐scale outbreak of the epidemic in relevant areas in China again. In addition, the global supply chain would continue to stagnate under the influence of the epidemic, which might cause great obstacles to the recovery and rebound of China's economy in the second quarter. At that time, the medical teams that have been withdrawn in batches will fight again, and cities will be closed again in different degrees. Some economic activities will be suspended, and the scene in the first quarter will be repeated, and even worse. The most serious period after the outbreak of the epidemic is during the Spring Festival holiday. The central government mobilized the whole country to assist Hubei Province, and guaranteed the supply of various medical and living materials to the greatest extent. If the epidemic comes again, we will also take measures to put out the threat of the virus again. However, the battle in half a year will have an inestimable long tail effect on China's economic growth in the second half of the year, and China's GDP in 2020 may probably show a negative growth state.

## 4 Summary

In this paper, based on the historical GDP data, this paper first estimates that China's GDP would reach 104 902 billion yuan in 2020 without the epidemic by using the neural network model. Second, we analyze the operation of various industries in the first quarter under the impact of the outbreak through the recent statistical data, and the calculation result shows that the GDP loss of China in the first quarter is about 4413.95 billion yuan. Then, taking June as the node, we analyze the different effects of three development scenarios of the global epidemic on China's economy and society. The first is an optimistic scenario. The global epidemic is effectively controlled in June. In order to achieve the tradition of “ensuring 6” of national economic growth, the GDP growth in the third and fourth quarters needs to reach 16.61%. The second scenario is that the overall domestic situation in June improves, while the situation of foreign epidemic is still severe, and China's import and export trade will be greatly affected. If the decline is 17.2%, the net export value in the third quarter of 2020 is estimated to be $98.6 billion due to the impact of the overseas epidemic. The prediction methods we used are relatively simple, and the results may be slightly different from the reality. The third is a pessimistic scenario. Affected by imported cases, China will have another outbreak in June, and the national economy will face a new round of stagnation challenges. Through the comparative analysis of these three scenarios, we need to clearly realize that the continuous and sufficient attention and support should be given for epidemic prevention and industrial development. In the current situation of more and more serious epidemic worldwide, China needs to take reasonable and effective measures in time. On the one hand, we need to stabilize the positive trend of domestic epidemic; on the other hand, we need to promote the gradual recovery and development of national economy.

First of all, we should stabilize the domestic epidemic situation, strengthen and control overseas import. According to the severity of the national epidemic situation, the government should implement scientific and dynamic measures. The entry personnel should be strictly subject to health and quarantine measures such as temperature detection, nucleic acid detection, and 14 days of medical observation and isolation. In the meanwhile, periodic disinfection treatment shall be carried out for transportation vehicles and goods. We should maintain the stability of the domestic epidemic situation as well as establish the international joint defense and control mechanism. Chinese enterprises can increase the production of medical protection materials and necessities, and provide necessary supply to other countries, which can not only improve China's production capacity, but also promote joint efforts to win the sniper war on epidemic prevention and control. Under the condition of stable domestic epidemic situation, all kinds of economic policies need to be implemented simultaneously, so as to promote enterprises to make up for losses and return to normal operation as soon as possible, and speed up the pace of economic recovery in China. This paper puts forward the following four macroeconomic policy recommendations:

First, the existing fiscal policies should be fully implemented and targeted fiscal and tax support policies should continue to be introduced in line with the trend of the epidemic and the economic recovery. The biggest problem for enterprises at present is the lack of cash flow, China has issued a series of fiscal and tax policies to help the enterprises in trouble, such as tax relief, reduction of pension medical payment, financial interest discount, and financial support. Among them, tax and fee reduction are mainly aimed at reducing the burden of enterprises. In order to support enterprises to repair capital flow as soon as possible, we should focus on fiscal policies, such as subsidies, tax rebates, special funds, transfer payment. Then, the deficit rate and expenditure can be further expanded. With the gradual improvement of the market economy, the fiscal policy can be adjusted in the aspect of stabilize growth, promote consumption and technology research of enterprises, and enhance the vitality of the economic market. At the same time, we should increase long‐term support for public health, social security and other areas related to people's livelihood, to enhance the ability of our people to resist risks.

Second, we should adopt more flexible monetary policies to ensure a reasonable and sufficient liquidity of the economy. The focus of monetary policy is to reduce interest rate and expand credit. The government and financial institutions should accurately identify the enterprises with good development prospects while facing temporary business difficulties, and provide them with targeted policy support such as reducing loan reserve, reducing benchmark interest rate and loan period extension, so as to reduce the financing cost of enterprises and enhance the availability of credit. Various financial tools should be made full use to expand the financing channels of enterprises.

Third, the government should comprehensively strengthen policies and measures to stabilize employment. Local governments should fully promote employment in accordance with local conditions. In the short term, we can accelerate the promotion of stable employment situation by providing special funds for employment, increase subsidies for job stabilization, provide employment guiding services, and improve the employment training system and other measures. Through the cooperation and assistance of local government and employers, the short‐term employment pressure can be relieved effectively. In the long term, the government can encourage effective investment, increase the market's ability to absorb labor and expand the total amount of social employment by optimizing the layout of industrial structure. By optimizing the industrial structure, we can reduce the employment pressure and unemployment risk.

Fourth, expand the scale of new infrastructure construction. Great efforts should be put to 5G, industrial Internet, Internet of things, artificial intelligence, digital economy and other emerging fields. By increasing the government's policy support in the field of new infrastructure, the social capital investment and the innovation and development of new technologies would be accelerated. Information technology should be made full use to upgrade traditional infrastructure, so as to open up more space for new consumption such as online entertainment, distance education and online e‐commerce, and promote industrial development and national economic growth.

## Conflict of Interest

The authors declare no conflict of interest.
